# Susceptibility of lung epithelium to neutrophil elastase: protection by native inhibitors

**DOI:** 10.1080/09629359791488

**Published:** 1997-12

**Authors:** L. Bingle, R. J. Richards, B. Fox, L. Masek, A. Guz, T. D. Tetley

**Affiliations:** 1 Department of Respiratory Medicine Imperial College School of Science Technology and Medicine at Charing Cross Hospital Fulham Palace Road London UK; 2 School of Molecular and Medical Biosciences University of Wales Cardiff Cardiff UK

## Abstract

The development of emphysema is thought to be due to an imbalance of proteases (especially neutrophil elastase [NE]) and antiproteases with loosening of the respiratory epithelium as an early event. We investigated the effect of NE on respiratory epithelial cell adherence in vitro , in the presence of varying concentrations and combinations of native inhibitors, α-1-proteinase inhibitor (PI) and secretory leukoprotease inhibitor (SLPI). SLPI was two to 12 times more effective than PI at preventing the effects of NE, especially when enzyme:inhibitor ratios were almost equivalent. Even when the concentration of SLPI was only 10% of the total (as in normal peripheral lung secretions), it gave greater protection than PI alone. This suggests that SLPI plays an important role in controlling neutrophil elastaseinduced inflammation and tissue damage.


Mediators of Inflammation, **6**, 345–354 (1997)

### Susceptibility of lung epithelium to neutrophil elastase: protection by native inhibitors

L. Bingle[1], R. J. Richards[2], B. Fox[1], L. Masek[1], A. Guz[1] and T. D. Tetley[1,CA]

[1]Department of Respiratory Medicine, Imperial College School of Science Technology and Medicine at Charing Cross Hospital, Fulham Palace Road, London; [2]School of Molecular and Medical Biosciences, University of Wales Cardiff, Cardiff, UK

[CA]Corresponding Author
Tel: (+44) 181 846 7182
Fax: (+44) 181 846 7170
Email: t.tetley@cxwms.ac.uk

**The development of emphysema is thought to be due to an imbalance of proteases (especially neutrophil elastase [NE]) and antiproteases with loosening of the respiratory epithelium as an early event. We investigated the effect of NE on respiratory epithelial cell adherence *in vitro*, in the presence of varying concentrations and combinations of native inhibitors, α-1-proteinase inhibitor (PI) and secretory leukoprotease inhibitor (SLPI). SLPI was two to 12 times more effective than PI at preventing the effects of NE, especially when enzyme:inhibitor ratios were almost equivalent. Even when the concentration of SLPI was only 10% of the total (as in normal peripheral lung secretions), it gave greater protection than PI alone. This suggests that SLPI plays an important role in controlling neutrophil elastase-induced inflammation and tissue damage.**

**Key words:** Alveolar, Bronchiolar, Epithelium, Matrix, Elastase, Antiprotease

### Introduction

Historically neutrophil elastase (NE) has been regarded as the primary mediator of tissue damage which occurs during pulmonary emphysema, notably by degradation of extracellular matrix.[1] More recent evidence suggests that NE activates certain metalloproteinases whilst inactivating their inhibitors[2,3] and that metalloproteinase levels are raised in emphysematous patients[4] suggesting that NE and metalloproteinases act in concert in the aetiology/pathology of this disease. In addition, NE has been shown to play a pro-inflammatory role in pulmonary inflammation involving neutrophil influx. For example, activation of complement to C5a[5] and stimulation of IL-8 synthesis and secretion by airway epithelial cells[6] would amplify neutrophil influx, whilst its ability to reduce airway epithelial cell ciliary beat frequency and secretagogue activity[7,8] would contribute to reduced clearance during diseases such as chronic bronchitis and cystic fibrosis. Thus NE activity is normally strictly regulated by systemic and locally produced inhibitors.

SLPI (12 kD) is a reversible inhibitor of NE, synthesized and secreted by non-ciliated airway and bronchiolar secretory cells;[9] SLPI will inhibit NE even when bound to a substrate.[10] In contrast, PI (52 kD) is the major serum-derived inhibitor of NE and transudes into the interstitium and airspaces of the lung. It complexes with, and irreversibly inhibits, free NE[10] as well as dissociating and inactivating NE bound to other, reversible, inhibitors e.g. SLPI,[11] prior to clearance of NE:PI complexes from the circulation. Elafin (12–14 kD) is synthesized by both Clara-like and alveolar type II pneumocyte-like cell lines;[12] the mechanism of action of elafin has not been completely characterized. Analysis of the antineutrophil elastase screen in human bronchoalveolar lavage shows that although PI forms a relatively high proportion of this screen[13] (except in PI deficiency, which predisposes to the development of emphysema), the other locally produced inhibitors are present at levels that suggest they also play a significant role in controlling the action of NE in epithelial lining fluid.[14] Like PI, SLPI and elafin may also vary in concentration between healthy individuals and according to the disease state of the lung.[13,15] Thus it seems likely that control of extracellular NE could depend not only on the relative levels and synchronized action of these different inhibitors but also on the levels and site of deposition of NE i.e. within the epithelial lining fluid or interstitium.

Induction of SLPI mRNA levels in primary human airway epithelial cells,[16] and retention of SLPI activity by proteolysed SLPI fragments following exposure to NE,[17] suggests that increased SLPI levels provide a sustained local

© 1997 Rapid Science Publishers 

anti-NE defence mechanism against excessive pulmonary NE activity. In addition, aerosolized rSLPI stimulates glutathione levels in sheep epithelial lining fluid[18] and downregulates IL-8 levels in respiratory secretions from patients with cystic fibrosis,[19] suggesting that SLPI has an important anti-inflammatory role in the lung.

Previous investigations comparing the ability of PI and SLPI to prevent neutrophil-mediated proteolysis of fibrinogen[20,21] and neutrophil-induced detachment of human umbilical endothelial cells[22] have shown that SLPI is a better inhibitor than PI when used at the same concentration but separate from each other. SLPI and PI were never used in combination, as is known to occur *in vivo*. Although it is likely that in the absence of sufficient antiprotease protection, NE also disrupts peripheral structural lung cell integrity during emphysema, there are no quantitative studies to illustrate this or to show how PI and SLPI, alone or combined, inhibit the effects of NE on peripheral lung cells.

Two important epithelial cells found in the area affected by emphysema, the respiratory bronchioles and alveolae, are the non-ciliated bronchiolar secretory cell (NCBSC) and type II pneumocyte respectively. Apart from producing SLPI, the NCBSC produces other proteins, such as surfactant apoproteins[23–25] and Clara cell secretory protein,[26] while a major function of the type II cell is to produce pulmonary surfactant,[27] the extracellular lining layer responsible for maintaining reduced surface tension and preventing alveolar collapse. Both cell types are involved in the metabolism of xenobiotics[28,29] as well as acting as progenitors to type I alveolar epithelial cells (type II[30]) and ciliated cells (NCBSC[31]). Damage to these epithelial cells could therefore have serious consequences in terms of lung defence and regeneration of damaged tissue. Adequate inhibition of NE during inflammatory cell influx to the respiratory units would therefore be critical in preventing the occurrence of emphysema and inflammatory lung disease processes.[32]

It is not possible to investigate the exact relationship between changes in NE load, inhibitor profile and lung tissue damage that may be relevant to the development of emphysema in an *in vivo* model. Consequently, we have adapted a model that was previously used to assess bronchiolar[33] and alveolar[34] toxicity *in vitro*, to study the effect of NE on respiratory epithelial cells under defined experimental conditions. The specific aim was to determine the effect of NE on pulmonary epithelial type II and NCBSC cell detachment from (i.e. damage), and their ability to adhere to (i.e. repair), extracellular matrix *in vitro*. The use of matrix was regarded as crucial as, *in vivo*, these cells would either be in contact with, or re-populate, extracellular matrix of either epithelial or endothelial origin. Therefore, matrices generated by both epithelial and endothelial cells were examined. As our previous studies on human bronchoalveolar lavage showed that the ratio of PI to SLPI varied,[13] the protective effect of PI and SLPI on NE-induced damage was also investigated when used alone and when combined in ratios representing that in normal bronchoalveolar lavage (PI:SLPI 9:1[13]) and bronchoalveolar lavage from smokers with obstructive lung disease (PI:SLPI 1:1[13]).

### Methods

### Activity of NE and antiproteases

The relative activity of commercially obtained NE (Elastin Products Comp. Inc., MO, USA) was assayed using the method of Nakajima *et al.*[35] The activity of PI (Sigma, A9024) and SLPI (purified in this laboratory as described previously[14]) was assessed against this NE and the molar concentration of active inhibitor could then be determined on the basis that PI and SLPI inhibit NE on a 1:1 molar basis.

These assays were carried out immediately before each experiment and inhibitory capacity was also assessed after incubation under experimental conditions at 37°C (without cells) for 18 h.

### Animals and removal of lungs for epithelial cell preparation

Male mice (BALB/C 25–30 g) and rats (WAG 250–300 g), bred in-house, were killed by a lethal intra-peritoneal injection of phenobarbitone/0.15 M saline (1:1 v/v) containing 300 units/ml heparin. The lungs were perfused free of blood by gravity feed of 0.15 M NaCl via the pulmonary artery and carefully removed intact from the cavity with a tracheal cannula still tied in place. The excised lungs were lavaged to remove free alveolar cells and pulmonary airway secretions (4 × 0·6 ml [mice] and 4 × 10 ml [rats] of sterile 0.15 M NaCl).

### Isolation of mouse NCBSC

NCBSC were isolated using the method of Oreffo *et al.*[36] The final cell sample was suspended in DCCM-1 medium (Biological Industries, Glasgow) containing 0.4% Ultroser G (Gibco, Paisley) and plated according to the protocol described below.

### Isolation of rat type II cells

Type II cells were isolated using the method of Richards *et al.*[37] The final cell pellet was processed in the same way as the NCBSC.

### Identification of NCBSC

Functional NCBSC may be distinguished from other cells present in the preparation by their high levels of nitroblue tetrazolium (NBT) reductase. The method of Oreffo *et al.*[36] was used to estimate the proportion of NCBSC present in the original isolate as well as those remaining after treatment with enzyme and inhibitors.

### Identification of type II cells

The type II cell is the only cell in the lung with appreciable levels of the enzyme alkaline phosphatase, this was therefore used to identify type II cells in the original cell isolate following the method of Miller *et al.*[38]

To estimate the number of cells remaining after enzyme/inhibitor treatment the cells were stained with Giemsa which has the advantage over the alkaline phosphatase stain which does not involve fixation and cannot be stored for long periods of time to enable counting.

### Experimental protocol

The experimental model was a modification of those described previously for toxicological studies on pulmonary alveolar and bronchiolar epithelial cells *in vitro*.[33,34] To mimic the *in vivo* conditions of adherence to extracellular matrix of endothelial origin, 96 well plates, in which endothelial cells were previously grown under controlled conditions, were used (Biological Industries). The cells are removed prior to purchase, leaving a coat of endothelial cell-derived extracellular matrix. This matrix is referred to as endothelial matrix in this study. It is similar in its composition to naturally occurring endothelial cell basement membrane which contains type IV collagen, laminin, heparan sulphate proteoglycan, fibronectin, entactin and nidogen. To mimic the *in vivo* situation of adherence to epithelial matrix, untreated 96 well plates were coated with Matrigel (Gibco, Paisley), an extracellular matrix of basement membrane synthesized by Engelbreth-Holm-Swarm mouse sarcoma consisting of laminin, type IV collagen, heparan sulphate proteoglycan, entactin and nidogen. This matrix is referred to as epithelial matrix in this study.

Cells were plated onto 96 well plates, $5 \times 10^4$ cells/100 ml DCCM-1 + 0.4% Ultroser G (media)/well, which were coated with either endothelial or epithelial matrix. Cells were studied either prior to adherence or following adherence, as follows:

(a) *adherent cells*, allowed to adhere for 16 h, the media removed, 100 ml of fresh media added to each well and exposed to NE and inhibitors for 2 h;
(b) *freshly plated cells*, treated with enzyme and inhibitors at the time of plating and incubated for 18 h.

### Effect of NE and inhibitors on cell adherence/detachment

Fifty ml of media containing 0, 2, 10, 20 and 40 pmol active inhibitor in each of the following combinations—PI alone, SLPI alone, 9 PI:1 SLPI and 1 PI:1 SLPI—were then added. Immediately after, 50 ml of media containing 0, 3, 6, 12 and 24 pmol active NE were added to each dose and each combination of inhibitor. Thus cells were incubated (i) alone, (ii) with all doses of NE, and (iii) with each dose of NE mixed with every dose and combination of inhibitor. After 2 (adherent cells) or 18 h (freshly plated cells; see above), the incubation media and any non-adherent or dislodged cells were removed. Non-adherent cells were assayed for viability with trypan blue, while adherent cells were stained and counted as described above. This experiment was performed in triplicate for each set of conditions on three separate occasions.

### Enumeration of cells

After staining, a representative tract of the culture was selected across the diameter of the well to account for any plating anomalies. Three different areas were counted along this tract (for control cultures this represents 800–1000 cells) and from this the mean number of cells/unit area could be ascertained. Within any one well the standard error of the mean, in control cultures, or those treated with the same dose of HNE (where applicable) did not exceed ±15%. The number of cells/unit area determined for the control culture was taken as 100% and the number of attached cells/unit area in the presence of elastase and/or inhibitors was expressed as a percentage of the control value.

All data obtained from these experiments were analysed using analysis of variance (BMDP Statistical Software, University of California).

### Release by NE of endothelial and epithelial matrix from tissue culture plastic

Matrix that is not populated by adherent cells may be susceptible to NE (e.g. in studies of non-adherent cells) and is likely to be degraded/ modified and contribute to changes in epithelial cell adherence. Inhibitors may modify the effect of NE on the matrix. Thus, both endothelial and epithelial matrix-coated plates were exposed to NE and inhibitors in an identical manner to that described above for non-adherent cells but in the absence of cells, to examine the effect of NE on the matrix alone. After 18 h, the medium was removed, the wells washed in PBS buffer and the remaining matrix protein level assayed *in situ* using the Bicinchoninic acid (BCA) protein assay as described in the manufacturer's instruction manual (Pierce, Rockford, Illinois, USA). Absorbance was measured at 562 nm on a Labsystems MCC340 plate reader and protein levels expressed as a percentage of the non-proteolysed control value.

### Results

### Cell isolation

The proportion of type II cells present in the original isolate was 95–99% whilst the proportion of NCBSC was 70–75%. Fig. 1 shows representative cultures, growing on endothelial matrix (type II, Fig. 1A; NCBSC, Fig. 1D).

### Effect of NE on ECM and matrigel

Elastase caused the release of endothelial matrix from tissue culture plastic in a dose-dependent manner; even 3 pmol of enzyme caused 35% degradation whilst 12 and 24 pmol caused over 80% degradation (Fig. 2). There was no release of epithelial matrix (data not shown).

### Effect of PI and SLPI on NE-induced endothelial matrix degradation

There was no loss of NE activity or inhibitory capacity of PI or SLPI incubated in tissue culture media for 18 h (data not shown). When endothelial matrix was incubated with low doses of NE, 10 pmol or more of PI or SLPI performed similarly and prevented, or significantly reduced, endothelial matrix degradation (Fig. 2). However, the effect of higher doses of NE could not be completely prevented by the addition of a molar excess of PI or SLPI, although SLPI was more effective.

### NE-induced detachment of adherent cells

Three pmol of NE did not induce epithelial cell detachment and no further studies were carried out with this dose of enzyme (data not shown). Although 6 pmol NE did not affect NCBSC or cause type II cell detachment from endothelial matrix, this dose of enzyme did cause type II cell detachment from epithelial matrix (~ 40% detachment, Fig. 3A). Furthermore, type II cells were more susceptible than NCBSC to NE-induced detachment from both matrices after exposure to 12 pmol NE ($P < 0{\cdot}001$). Twenty-four pmol NE had a similar effect on both cell types causing 70–80% detachment from both matrices.

Thus, unlike type II cells, detachment of NCBSC was not differentially affected by the matrix to which they adhered but was related to dose (Fig. 3A). This effect of NE is illustrated in Fig. 1.

### Effect of NE on cell adherence of freshly plated cells

The effect of NE on the adhesion of freshly plated cells is shown in Fig. 3B. Since the initial study with adherent cells showed that 6 pmol NE had little effect, the effect of this dose of enzyme was not examined.

The adherence of freshly plated type II cells on to endothelial matrix was reduced to 33% and 7% of untreated cells when exposed to 12 and 24 pmol NE respectively, while the adherence of NCBSC was virtually abolished (Fig. 3B). The effect of 12 pmol NE on type II cell adherence to epithelial matrix was similar to that seen on endothelial matrix (28% of control). However, NCBSC adherence to epithelial matrix was less affected (Fig. 3B). The effect of 24 pmol NE on the adherence of both cell types to epithelial matrix was similar (~ 12% of control).

### Differential effect of NE on freshly plated compared with adherent epithelial cells

The effects of 24 pmol NE on adherence of freshly plated cells was more marked than that on cell detachment; thus in every equivalent combination of cell type, matrix and dose of enzyme, proportionally more freshly plated cells

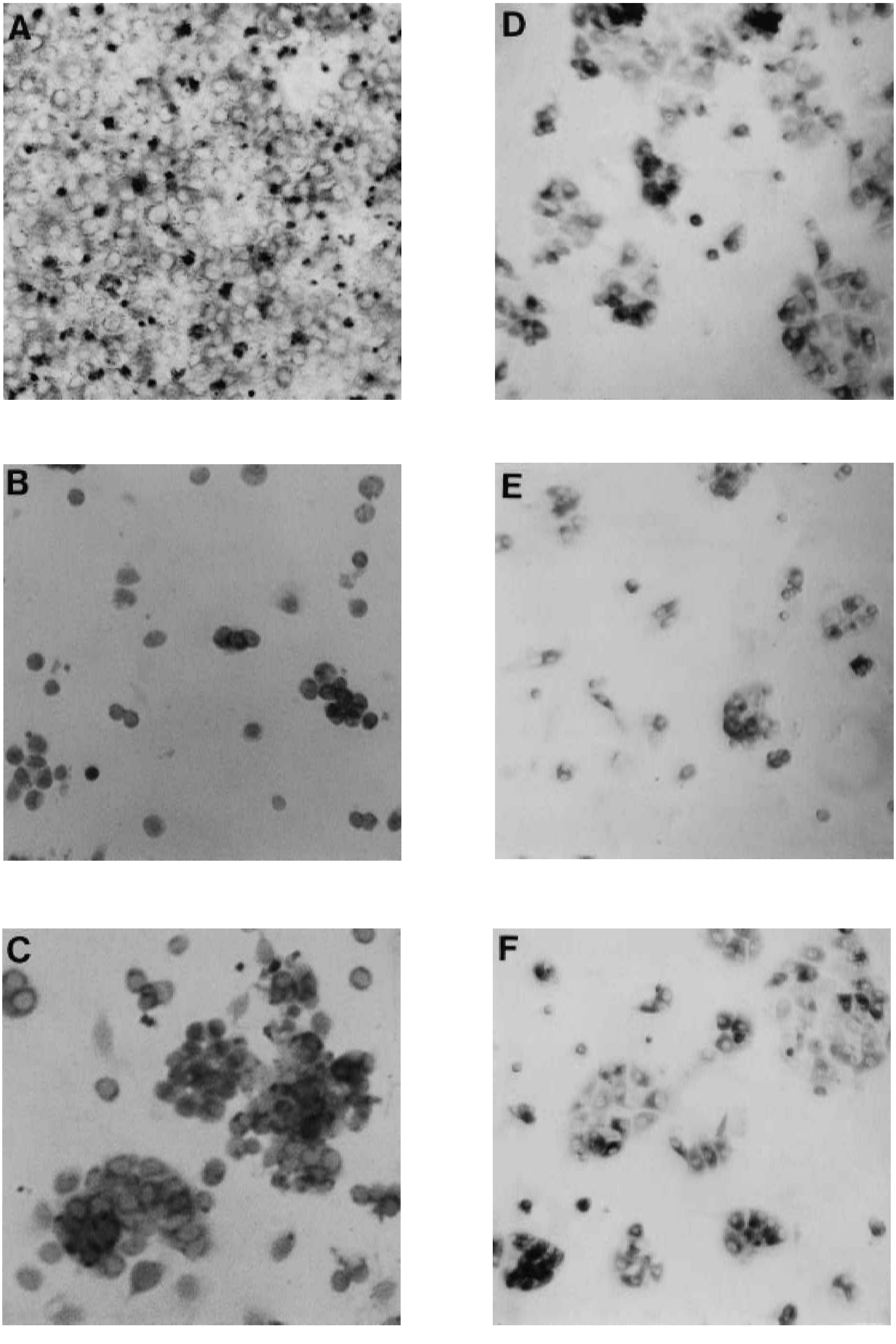

FIG. 1. Adherent alveolar type II cells (A–C) and NCBSC (D–F). Before treatment (A, D); after treatment with 24 pmol NE (B, E); after concurrent treatment with 24 pmol NE and 20 pmol PI (C, F).

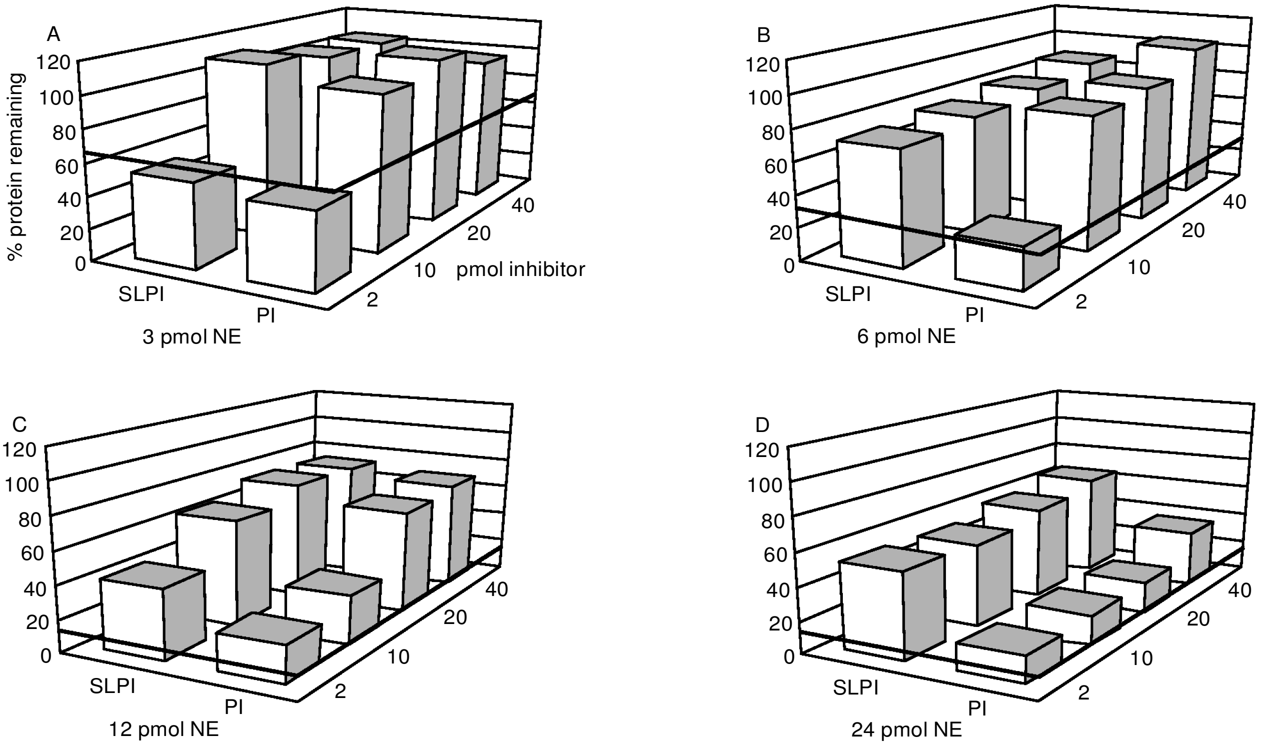

FIG. 2. The effect of incubation of endothelial matrix-coated wells with NE. The solid line represents the proteolytic effect of NE alone, while the bars represent NE in the presence of SLPI or PI.

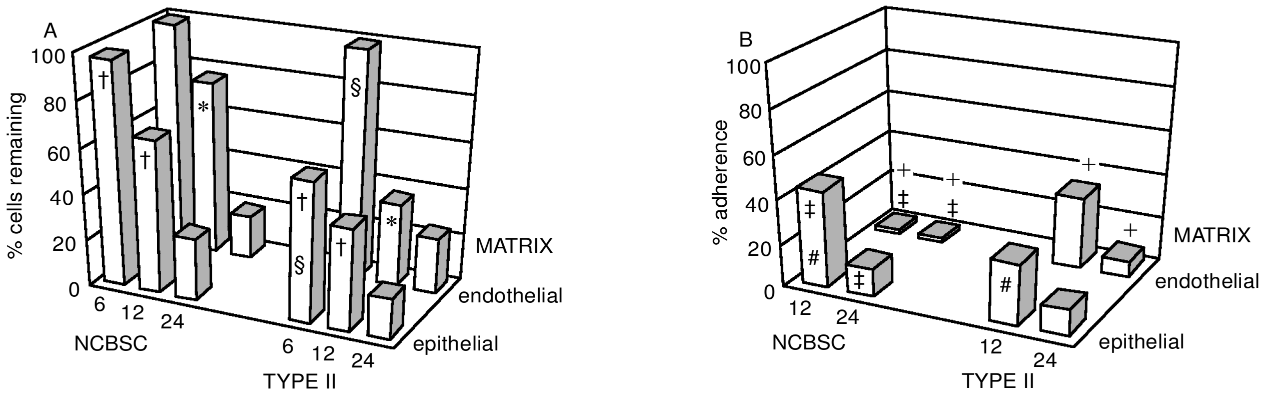

FIG. 3. The effect of 6, 12 and 24 pmol NE on cell detachment from (A), or cell adherence to (B), endothelial matrix and epithelial matrix. (A)* = 12 pmol NE/endothelial matrix; NCBSC detachment < type II cells, $P < 0{\cdot}001$; † = 6 and 12 pmol NE/epithelial matrix; NCBSC detachment < type II cells, $P < 0{\cdot}01$; § = 6 pmol NE/type II cell detachment; endothelial matrix < epithelial matrix, $P < 0{\cdot}001$. (B) ‡ = 12 and 24 pmol NE/NCBSC adherence; endothelial matrix < epithelial matrix, $P < 0.001$; + = 12 and 24 pmol NE/endothelial matrix: NCBSC adherence < type II cells, $P < 0.01$; # = 12 pmol NE/epithelial matrix; type II cell adherence < NCBSC, $P < 0.001$.

were prevented from adhering than were displaced by NE (Fig. 3).

### Prevention of NE-induced changes in cell adherence by PI and SLPI

Two pmol of inhibitor had a negligible effect on NE-induced cell attachment or detachment. The effect of 6 pmol of NE was efficiently prevented by all levels and combinations of inhibitors (data not shown). The effect of inhibitors on 12 and 24 pmol NE were similar, and related to the ratio of enzyme to inhibitor. Consequently the data for 24 pmol NE is presented as this allows discussion of a wider range of inhibitor to enzyme ratios. The protective effects of inhibitor are illustrated pictorially in Fig. 1.

### Prevention of NE-induced cell detachment by PI and SLPI

*Endothelial matrix*. Forty pmol of any combination of inhibitor prevented the effects of 24 pmol NE on type II cell detachment. Addition of approximately equimolar concentrations of inhibitor (i.e. 24 pmol NE: 20 pmol inhibitor) partially prevented NE-induced type II cell detachment. This effect was positively related to the level of SLPI, SLPI being approximately 1.5 times as effective as PI alone (Fig. 4A). A small but significant effect on NE-induced type II cell detachment was seen with 10 pmol inhibitor.

In contrast, there was no difference between PI and SLPI in their ability to prevent NE-induced NCBSC detachment from endothelial matrix. The reduction in cell detachment in the presence of inhibitor was directly related to inhibitor level, such that with 10 pmol of inhibitor only 50% (compared with 20%) were dislodged, while there was no cell detachment with 40 pmol of inhibitor (Fig. 4B).

*Epithelial matrix*. Forty pmol of any combination of inhibitor prevented NE-induced cell detachment of both type II and NCBSC cells. The action of 24 pmol NE on both cell types could be inhibited by 20 pmol of inhibitor, inhibition being more effective with increasing SLPI to PI ratio (SLPI 1.5–2 times more effective than PI, Fig. 4C,D). Interestingly a similar inhibitory pattern was observed when NCBSC cells were exposed to 24 pmol NE and 10 pmol inhibitor.

### Prevention of the effects of NE on epithelial cell adherence by PI and SLPI

*Endothelial matrix*. The effects of 24 pmol NE on either cell type could not be completely prevented by 20 or 40 pmol of either inhibitor despite their ability to inhibit NE on a 1:1 molar basis in the absence of cells (Fig. 5A). Forty pmol PI significantly prevented the effects on type II cell adherence, allowing adherence to increase from 7% to 50% of the unexposed control level. However, 40 pmol SLPI was more effective, causing a reversal that achieved adherence that was 89% of unexposed control levels (Fig. 5A). A similar but less effective pattern on NE inhibition was observed with NCBSC exposed to 24 pmol NE. Thus, although there was no improvement in adherence of NCBSC after addition of 40 pmol PI, 40 pmol SLPI allowed 51% of the control adherence level. Inhibition was nevertheless

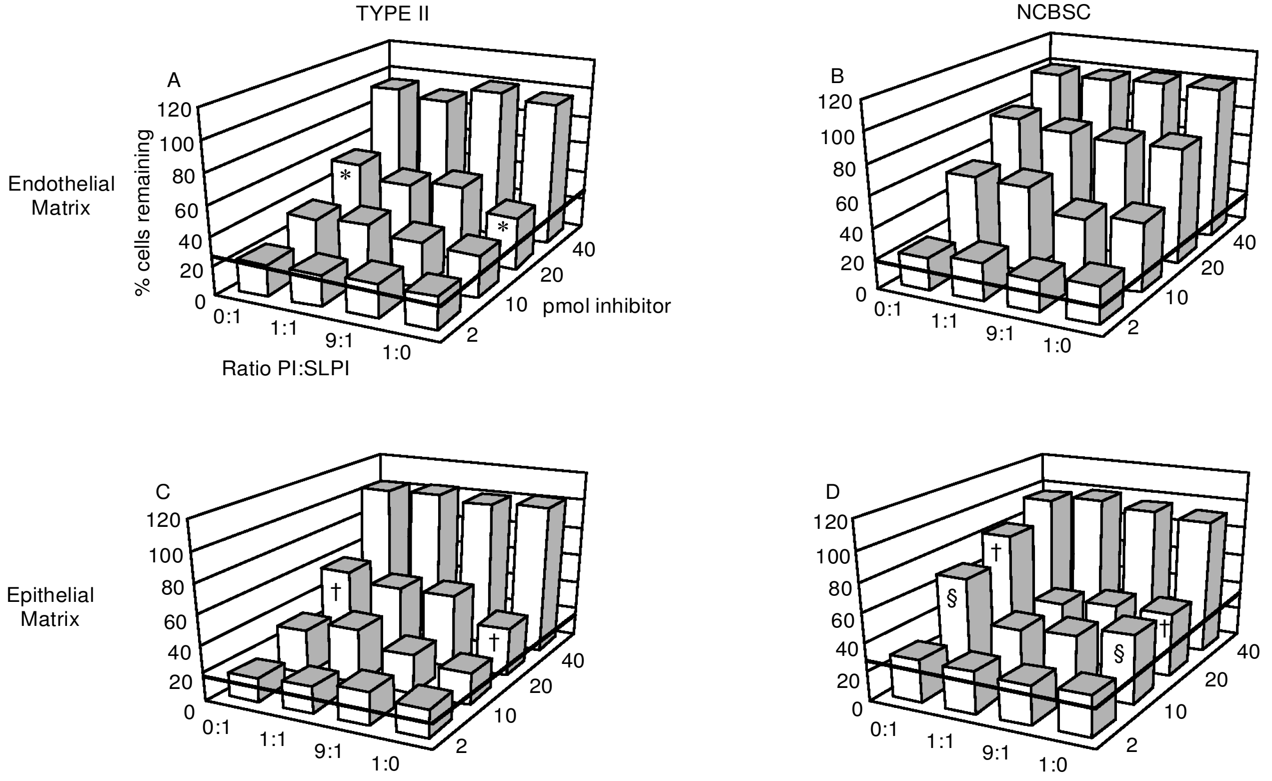

FIG. 4. Comparison between the inhibitory effect of PI and SLPI on 24 pmol NE and the detachment of adherent cells from endothelial matrix and epithelial matrix. The solid line represents NE alone while the bars represent NE in the presence of inhibitor. * = significantly enhanced inhibition of the action of NE on type II cell adherence to endothelial matrix by SLPI compared with PI; inhibitor level 20 pmol, $P < 0.01$; † = significantly enhanced inhibition of the action of NE on epithelial cell adherence to epithelial matrix by SLPI compared with PI; inhibitor level 20 pmol, $P < 0.001$; § = significantly enhanced inhibition of the action of NE on NCBSC adherence to epithelial matrix by SLPI compared with PI; inhibitor level 10 pmol, $P < 0.01$.

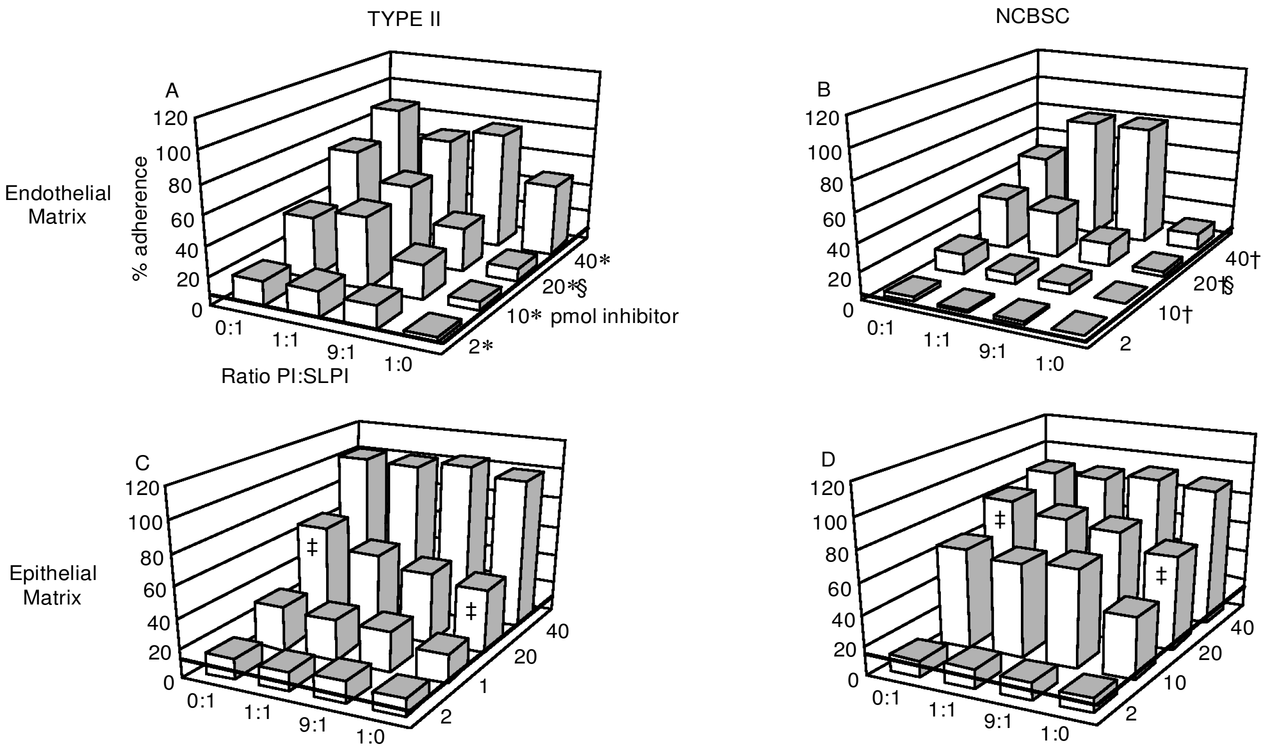

FIG. 5. Comparison between the inhibitory effect of PI and SLPI on 24 pmol NE and the ability of freshly plated cells to adhere to endothelial matrix and epithelial matrix. The solid line represents NE alone while the bars represent NE in the presence of inhibitor. * = significant prevention of the effect of NE on type II cell adherence to endothelial matrix by either PI or SLPI; inhibitor level—40 pmol ($P < 0.001$), 20 pmol ($P < 0.001$, excepting PI alone), 10 pmol ($P < 0.001$, excepting PI alone) and 2 pmol ($P < 0.05$, excepting PI alone); † = significant prevention of the effect of NE on NCBSC adherence to endothelial matrix by PI combined with SLPI; inhibitor level 40 pmol ($P < 0.001$), 20 pmol ($P < 0.001$) and 10 pmol ($P < 0.05$, SLPI alone); § = significantly enhanced inhibition of the action of NE on epithelial cell adherence to endothelial matrix by SLPI compared with PI; inhibitor level 20 pmol, $P < 0.001$; ‡ = significantly enhanced inhibition of the action of NE on epithelial cell adherence to epithelial matrix by SLPI alone compared with PI alone: inhibitor level 20 pmol, $P < 0.05$.

incomplete. For both cell types, the most effective reversal of the effect of NE occurred when SLPI was present. This was most noticeable when there were almost equimolar levels of NE and inhibitor, the efficacy of the inhibitor increased with increasing levels of SLPI, SLPI being two to 12 times more effective at preventing the effects of NE than PI (Fig. 5A,B).

*Epithelial matrix.* In contrast to cell adherence to endothelial matrix, the effect of 24 pmol of NE on both cell types was almost completely prevented by 40 pmol of any combination of inhibitor whilst the lower doses of inhibitor were also significantly more effective at preventing the effects of 24 pmol NE in this situation (Fig. 5). Once again at equimolar concentrations of NE with inhibitor, SLPI was more effective (approximately 1.5 times).

### Discussion

Mediators released by neutrophils are thought to make a significant contribution to tissue destruction; NE, in particular, has been shown to play an important role in the initiation and perpetuation of an inflammatory response. Studies in experimental animals suggest that loosening of the respiratory epithelium may be an early event in the development of emphysema.[39] We have shown that this could result from release of excessive NE by neutrophils at the apical pulmonary respiratory epithelium. Although there have been numerous studies on the effect of NE on airway epithelial cells[7,40] little is known about its effect on the respiratory epithelium. In this study we examined the effect of NE on adherent respiratory epithelial cells (type II and NCBSC), representing resident epithelial cells *in situ*, and non-adherent cells, representing dislodged epithelial cells *in situ*. Dislodged or loosened cells may exist as a result of previous protease action, oxidant action, due to the cytotoxic effects of cigarette smoke, or for other, unknown reasons.

As expected, when adherent cells were exposed to NE, dose-dependent detachment of both cell types was observed, although the degree of detachment was greater with type II cells than with NCBSC. In addition, the effect of

NE on adherent NCBSC was independent of the source of the extracellular matrix, whereas type II cells were more susceptible to NE-induced detachment from epithelial matrix than from endothelial matrix. This suggests that, *in situ*, NCBSC are less likely to be displaced by NE than alveolar type II cells, which are most vulnerable when in contact with epithelial cell-derived matrix.

In contrast, the effect of NE on cell adherence of freshly plated cells was more marked and suggests that alveolar type II cells would be more likely than NCBSC to 're-populate' denuded, exposed matrix. Since NE degrades endothelial matrix (possibly reflecting degradation of the fibronectin component) and NCBSC do not adhere to tissue culture plastic, it seems possible that susceptibility of extracellular matrix to NE and preference of NCBSC for a specific matrix may exist *in vivo* and hence be important in repopulation and repair mechanisms. Furthermore, NE may be acting on cell membrane proteins, such as adhesion molecules, since both type II cell and NCBSC adherence to epithelial matrix was inhibited by NE, even though the epithelial matrix used in this study was not significantly solubilized by NE under these experimental conditions.

Protection, by SLPI, of epithelial cells from NE-induced damage, was equal to, or better than, the serum-derived inhibitor, PI, and was most noticeable when the enzyme:inhibitor ratios were almost equivalent, a situation that might occur in the microenvironment of the phagocyte *in vivo*. Indeed, PI was often a poor inhibitor of the effects of NE, particularly on adherence of freshly plated cells to endothelial matrix. This probably reflects two factors—endothelial matrix is proteolysed by NE and PI cannot inhibit NE bound to extracellular matrix.[10,32] Thus, prior to cell adherence, NE probably bound rapidly to endothelial matrix and, having done so, could only be inhibited by SLPI (SLPI is believed to bind to extracellular matrix and can inhibit extracellular matrix-bound NE).[10,41] This explanation is supported by the observation that, when studied in the absence of cells, more endothelial matrix was found to be solubilized by NE in the presence of PI than SLPI. Such complex interactions may explain why a molar excess of inhibitor would not always prevent NE-induced epithelial cell damage. Reduced levels or activity of SLPI *in vivo* (which is released both apically and basally, into the interstitial extracellular matrix, by secretory epithelial cells[42]) might mean that NE that has bound to extracellular matrix cannot be sufficiently inhibited by other inhibitors present within the lung tissue and hence that tissue proteolysis will progress. This is supported by a recent study[43] which showed a negative correlation between elevated NE activity and SLPI levels in sputum from patients who had exacerbative chronic obstructive pulmonary disease, suggesting that the condition was worse when SLPI levels were reduced. There was no correlation between PI levels, NE activity and respiratory symptoms, possibly highlighting the significance of SLPI in controlling the action of NE and severity of disease.

It is interesting that adherent NCBSC were markedly more resistant to NE than non-adherent NCBSC and also that the difference between the protective effects of PI and SLPI were less noticeable following epithelial cell adherence. As the cells had already adhered, less extracellular matrix or cell surface molecules would be exposed to NE and hence less 'protection' would be required from the inhibitors. PI would be more likely to inhibit unbound NE, having a higher $K_{ass}$ than SLPI for free NE.[11,44] Synthesis and secretion of SLPI by the adherent NCBSC may also provide increased protection from NE, although analysis of NCBSC culture supernatant with antibodies to human SLPI was negative (data not shown). However, this may reflect lack of cross-reactivity between species.

The data suggest that even when the proportion of SLPI in the antiprotease mixture added to the cells is only 10% of the total (cf. normal lung ratios[13,15]), the degree of protection from NE-induced damage is increased above that of PI alone. Since those with bronchitis and emphysema may have even higher proportions of SLPI (50%[13]) and in the present study a 1:1 ratio of PI to SLPI conferred greater protection from NE-induced damage, one could hypothesize that increased production of SLPI by the pulmonary epithelium gives extra protection and may be a defence mechanism rather than a non-specific result of hypersecretion.

In conclusion, this study suggests that incomplete inhibition of NE released at the apical surface of respiratory epithelium may contribute to loosening and possibly detachment of the epithelium. The magnitude of the effect is likely to depend on both the nature of the underlying matrix (i.e. susceptibility to NE) and the differential action of the enzyme on epithelial cells from bronchiolar and alveolar regions of the respiratory unit, including adhesion molecules. Weak epithelial cell adhesion and cell detachment is likely to facilitate access of enzyme to the underlying interstitium and extracellular matrix, including basement membrane and elastic tissue, which are susceptible to proteolysis by NE. Such mechanisms may contribute to the tissue damage that occurs during the development of emphysema. Finally, this study shows that SLPI, particularly in combination with PI, plays a major role in protecting lung cells and interstitial components from the destructive action of NE. We hypothesize that reduced levels or activity of SLPI *in vivo* may contribute to NE-induced lung damage and neutrophil-mediated lung disease.

### References

1. Gadek JE, Fels GE, Zimmerman RL, Rennard SI, Crystal RG. Antielastases of the human alveolar structures. Implications for the protease-antiprotease theory of emphysema. *J Clin Invest* 1981; **68**: 889–898.
2. Palmgren MS, deShazo RD, Carter RM, Zimny ML, Shah SV. Mechanisms of neutrophil damage to human alveolar extracellular matrix: the role of serine and metalloproteases. *J Allergy Clin Immunol* 1992; **89**: 905–915.
3. Okada Y, Watanabe S, Nakanishi I, *et al*. Inactivation of tissue inhibitor of metalloproteinases by neutrophil elastase and other serine proteinases. *FEBS Letts* 1988; **229**: 157–160.
4. Finlay GA, Russell KJ, McMahon KJ, *et al*. Elevated levels of matrix metalloproteinases in bronchoalveolar lavage fluid of emphysematous patients. *Thorax* 1997; **52**: 502–506.
5. Weiss SJ. Tissue destruction by neutrophils. *New Engl J Med* 1989; **320**: 365–376.
6. Nakamura H, Yoshimura K, McElvaney NG, Crystal RG. Neutrophil elastase in respiratory epithelial lining fluid of individuals with cystic fibrosis induces interleukin 8 gene expression in a human bronchial epithelial cell line. *J Clin Invest* 1992; **89**: 1478–1484.
7. Amatini R, Wilson R, Rutman A, *et al*. Effects of human neutrophil elastase and *Pseudomonas aeruginosa* proteinases on human respiratory epithelium. *Am J Respir Cell Mol Biol* 1991; **4**: 26–32.
8. Sommerhof CP, Nadel JA, Basbaum CB, Caughey C. Neutrophil elastase and cathepsin G stimulate secretion from cultured bovine airway gland serous cells. *J Clin Invest* 1990; **85**: 682–689.
9. Water R, Willems LNA, van Muyen GNP, *et al*. Ultrastructural localisation of bronchial antileukoprotease in central and peripheral human airways by a gold-labelling technique using monoclonal antibodies. *Am Rev Respir Dis* 1986; **133**: 882–890.
10. Bruch M, Bieth JG. Influence of elastin on the inhibition of leucocyte elastase by α-1-proteinase inhibitor and bronchial inhibitor. *Biochem J* 1986; **238**: 269–273.
11. Gauthier F, Fryksmark U, Ohlsson K, Bieth JG. Kinetics of the inhibition of leucocyte elastase by the bronchial inhibitor. *Biochem Biophys Acta* 1982; **700**: 178–183.
12. Sallenave JM, Silva A, Marsden ME, Ryle RP. Secretion of mucus proteinase inhibitor and elafin by a Clara cell and a type II pneumocyte cell line. *Am J Resp Cell Mol Biol* 1993; **8**: 126–133.
13. Tetley TD, Smith SF, Burton GH, Winning AJ, Cooke NT, Guz A. Effects of cigarette smoking and drugs on respiratory tract proteases and antiproteases. *Eur J Respir Dis* 1987; **71**(S153): 93–102.
14. Smith SF, Guz A, Burton GH, Cooke NT, Tetley TD. Acid-stable low molecular mass proteinase inhibitors in human lung lavage. *Biol Chem Hoppe-Seyler* 1986; **367**: 183–189.
15. Stockley RA, Morrison HM. Elastase inhibitors of the respiratory tract. *Eur Resp J Suppl* 1990; **9**: 9s–15s.
16. Abbinante-Nissen JM, Simpson LG, Leikauf GD. Neutrophil elastase increases secretory leukoprotease inhibitor transcript levels in airway epithelial cells. *Am J Physiol* 1993; **265**: L286–L292.
17. Masuda K, Suga T, Takeuchi A, Kanesaki M, Imaizumi A, Suzuki Y. Specific cleavage of secretory leukoprotease inhibitor by neutrophil elastase and saliva. *Biochem Pharmacol* 1994; **48**: 651–657.
18. Gillesen A, Birrer P, McElvaney NG, *et al*. Recombinant secretory leukoprotease inhibitor augments glutathione levels in lung epithelial lining fluid. *J Appl Physiol* 1993; **75**: 825–832.
19. McElvaney NG, Nakamura H, Birrer P, *et al*. Modulation of airway inflammation in cystic fibrosis. *J Clin Invest* 1992; **90**: 1296–1301.
20. Stolk J, Davies P, Kramps JA, *et al*. Potency of antileukoprotease and α-1-antitrypsin to inhibit degradation of fibrinogen by adherent polymorphonuclear leucocytes from normal subjects and patients with chronic granulomatous disease. *Am J Res Cell Mol Biol* 1992; **6**: 521–526.
21. Llewellyn-Jones CG, Lomas DA, Stockley RA. Potential role of recombinant secretory leucoprotease inhibitor in the prevention of neutrophil-mediated matrix degradation. *Thorax* 1994; **49**: 567–572.
22. Hiemstra PS, Kramps JA, de Vreede TM, Breedveld FC, Daha MR. Inhibition of polymorphonuclear leukocyte-mediated endothelial cell detachment by antileukoprotease: a comparison with other proteinase inhibitors. *Immunobiology* 1991; **182**: 117–126.
23. Auten RL, Watkins RH, Shapiro DL, Horowitz S. Surfactant apoprotein A is synthesised in airway cells. *Am J Resp Cell Mol Biol* 1990; **3**: 491–496.
24. Crouch E, Parghi D, Kuan SF, Persson A. Surfactant protein D: subcellular localisation in non-ciliated bronchiolar epithelial cells. *Am J Physiol* 1992; **263**: L60–L66.
25. Phelps DS, Floros J. Localisation of surfactant protein synthesis in human lung by in situ hybridisation. *Am Rev Respir Dis* 1988; **137**: 939–942.
26. Singh G, Singh J, Katyal SL, *et al*. Identification, cellular localisation, isolation and characterisation of human Clara cell 10 kD protein. *J Histochem Cytochem* 1988; **36**: 73–80.
27. Macklin CC. The pulmonary alveolar mucoid film and the pneumocyte. *Lancet* 1954; **1**: 1099–1104.
28. Devereux TR. Alveolar type II and Clara cells: isolation and xenobiotic metabolism. *Environ Health Persp* 1984; **56**: 95–101.
29. Devereux TR, Fouts JR. Xenobiotic metabolism by alveolar type II cells isolated from rabbit lung. *Biochem Pharmacol* 1981; **30**: 1231–1237.
30. Adamson IYR, Bowden DH. The type 2 cell as progenitor of alveolar epithelial regeneration. *Lab Invest* 1974; **30**: 35–42.
31. Evans MJ, Shami SG, Cabral-Andersen LJ, Dekker N. Role of non-ciliated cells in the renewal of the bronchiolar epithelium of rats exposed to $NO_2$. *Am J Pathol* 1986; **123**: 126–133.
32. Bingle L, Tetley TD. Secretory leukoprotease inhibitor: partnering α1-proteinase inhibitor to combat pulmonary inflammation. *Thorax* 1996; **51**: 1273–1274.
33. Richards RJ, Oreffo VIC, Lewis RW. Clara cell cultures from the mouse and their reaction to bronchiolar toxins. *Env Health Persp* 1990; **85**: 119–127.
34. Jones HE, Blundell GK, Tidwell RR, Hall JE, Farr SJ, Richards RJ. The accumulation of pentamidine and the toxic effects of the drug, its selected analogues and metabolites on isolated alveolar cells. *Toxicology* 1993; **80**: 1–12.
35. Nakajima K, Powers JC, Ashe BM, Zimmerman M. Mapping the extended substrate binding site of cathepsin G and human leucocyte elastase. *J Biol Chem* 1979; **254**: 4027–4032.
36. Oreffo VIC, Morgan A, Richards RJ. Isolation of Clara cells from the mouse lung. *Env Health Persp* 1990; **85**: 51–64.
37. Richards RJ, Davies N, Atkins J, Oreffo VIC. Isolation, biochemical characterisation and culture of lung type II cells of the rat. *Lung* 1987; **165**: 143–158.
38. Miller BE, Chapin RE, Pinkerton KE, Gilmore LB, Maronpot RR, Hook GR. Quantitation of silica-induced type II cell hyperplasia by using alkaline phosphatase histochemistry in glycol methacrylate embedded lung. *Exp Lung Res* 1987; **12**: 135–148.
39. Snider G, Lucey EC, Stone PJ. Animal models of emphysema. *Am Rev Respir Dis* 1986; **133**: 149–169.
40. Rickard KA, Taylor J, Rennard SI. Observations of development of resistance to detachment of cultured bovine bronchial epithelial cells in response to protease treatment. *Am J Respir Cell Mol Biol* 1992; **6**: 414–420.
41. Rice WG, Weiss SJ. Regulation of proteolysis at the neutrophil-substrate interface by secretory leukoprotease inhibitor. *Science* 1990; **249**: 178–181.
42. Dupuit F, Jacquot J, Spilmont C, Tournier JM, Hinnrasky J, Puchelle E. Vectorial delivery of newly-synthesised secretory proteins by human tracheal gland cells in culture. *Epith Cell Biol* 1993; **2**: 91–99.
43. Piccioni PD, Kramps JA, Rudolphus A, Bulgheroni A, Luisetti M. Proteinase/proteinase inhibitor imbalance in sputum sol phases from patients with chronic obstructive pulmonary disease. *Chest* 1992; **102**: 1470–1476.
44. Beatty K, Bieth J, Travis J. Kinetics of association of serine proteinases with native and oxidised α-1-proteinase inhibitor and α-1-chymotrypsin. *J Biol Chem* 1980; **255**: 3931–3934.

ACKNOWLEDGEMENTS. The authors would like to thank Dr K. MacRae for his help with the statistics in this paper, Ian Witherden for his assistance in the production of Fig. 1 and Dr K. Murphy for his assistance in the production of Figs 2–5. This work was supported by the Medical Research Council, UK.

**Received 1 July 1997;**
**accepted in revised form 28 August 1997**
